# Utilization of silicon nanowire field-effect transistors for the detection of a cardiac biomarker, cardiac troponin I and their applications involving animal models

**DOI:** 10.1038/s41598-020-78829-7

**Published:** 2020-12-16

**Authors:** Shih-Mein Chang, Sathyadevi Palanisamy, Tung-Ho Wu, Chiao-Yun Chen, Kai-Hung Cheng, Chen-Yi Lee, Shyng-Shiou F. Yuan, Yun-Ming Wang

**Affiliations:** 1 Department of Biological Science and Technology, Institute of Molecular Medicine and Bioengineering, Center for Intelligent Drug Systems and Smart Bio-Devices (IDS2B), National Chiao Tung University, 75 Bo-Ai Street, Hsinchu, 300 Taiwan, ROC; 2 Division of Cardiovascular Surgery, Department of Surgery and Division of Surgical Critical Care, Department of Critical Care Medicine, Veterans General Hospital, Kaohsiung, 813 Taiwan, ROC; 3 Department of Radiology, Faculty of Medicine, College of Medicine, Kaohsiung Medical University, Kaohsiung, Taiwan, ROC; 4 Department of Medical Imaging, Kaohsiung Medical University Hospital, Kaohsiung, Taiwan, ROC; 5 Division of Cardiology, Department of Internal Medicine, Kaohsiung Medical University Hospital, Kaohsiung, Taiwan, ROC; 6 Department of Electronics Engineering, National Chiao Tung University, Hsinchu, Taiwan, ROC; 7 Translational Research Center, Kaohsiung Medical University Hospital, Kaohsiung Medical University, Kaohsiung, Taiwan, ROC; 8 Department of Obstetrics and Gynecology, Kaohsiung Medical University Hospital, Kaohsiung Medical University, Kaohsiung, Taiwan, ROC; 9 Faculty and College of Medicine, Kaohsiung Medical University, Kaohsiung, Taiwan, ROC; 10 Department of Biomedical Science and Environmental Biology, Center for Cancer Research, Kaohsiung Medical University, Kaohsiung, 807 Taiwan, ROC

**Keywords:** Biomarkers, Medical research, Nanoscience and technology

## Abstract

This study develops an ultrasensitive electrical device, the silicon nanowire-field effect transistor (SiNW-FET) for detection of cardiac troponin I (cTnI) in obesity induced myocardial injury. The biosensor device utilizes metal–oxide–semiconductor (MOS) compatible top-down methodology for the fabrication process. After fabrication, the surface of the SiNW is modified with the cTnI monoclonal antibody (Mab-cTnI) upon covalent immobilization to capture cTnI antigen. The sensitivity of the device is also examined using cTnI at different concentrations with the lowest detection limit of 0.016 ng/mL. The electrocardiogram (ECG), magnetic resonance imaging (MRI), and superior vena cave (SVC) provide more information about cardiac responses in a mouse model of acute myocardial infarction (AMI). Further, magnetic resonance imaging helps to evaluate the cardiac output of an obesity induced myocardial injury mouse model. These methods play an essential role in monitoring the obesity based cardiac injury and hence, these studies were carried out. This is the first report to use the ECG, MRI, and SVC sampling methods to study the obesity based cardiac injury involving Syrian hamsters as animal models. The proposed SiNW-FET in this study shows greater sensitivity than the previously developed devices and demonstrates great potential for future applications in point-of-care (POC) diagnosis.

## Introduction

Cardiovascular diseases are major causes of death worldwide, which continue to grow due to aging^1,2^. The AMI is a prevalent cardiovascular disease diagnosed by detection of cardiac biomarkers including myoglobin, creatinine kinase-MB, cardiac troponin I (cTnI), and cardiac troponin T (cTnT). Among them, cTnI and cTnT are more selective and sensitive^3^. When AMI occurs, they are released by dying cardiomyocytes within 2–4 h, peak around 1–2 days and remain in the bloodstream over 10 days^4,5^. The limitation of conventional cTn assays is their low sensitivity and delayed increase in circulation for 6–9 h^6^. Both high-sensitivity cTnI (hs-cTnI) and high-sensitivity cTnT (hs-cTnT) provide high diagnostic and prognostic accuracy for AMI^7^. However, hs-cTnI have a higher diagnostic accuracy in early presenters (3 h since chest pain onset)^8^.

In addition, hs-cTn is recognized as a sensitive marker for subclinical myocardial injury^9,10^, which is frequently associated with obesity^11^. Obesity is a prevalent chronic disease worldwide^12^ and constitutes an independent risk circumstance for heart failure, coronary artery disease, diabetes mellitus, and hypertension^13,14^. Long-standing obesity causes left ventricular hypertrophy and systolic and diastolic dysfunctions^15^. Hence, early detection of obesity-associated cardiac changes is essential to prevent eventual heart failure.

Ultrasensitive and label-free detection has been achieved by using various transduction methodologies^16–18^. One such strategy used in electrical biosensor scaffolds is based on the capture and detection of biomarkers by materials coated on the electrode surface which converts the biological signal into a quantifiable electrical signal response^19^. Among various nanomaterials for biosensing, silicon nanowire (SiNW) is widely used for the detection of biomolecular strength changes which affect the electrical properties of the device. Silicon nanowire-field effect transistors (SiNW-FETs) provide label-free and real-time detection of various biomarkers^20–23^. These devices are constructed using microfabrication technology which relies on the binding of antibody and antigen on the nanowire surface. The first SiNW device was developed as a pH sensor in 2001 and the same strategy has been used for the detection of proteins, viruses and DNA molecules in the following years^24^. However, much less attempt has been made for the detection of obesity induced myocardial injury.

Although numerous improvements were developed in the treatments of patients with AMI and myocardial injury, the occurrence of heart failure after cardiac injuries continues to be high and the survival rate remains poor^25^. Some of the FETs has reported the detection limit of cTnI around 100 pg/mL, which is not efficient for the diagnosis of AMI^23,26^.

Magnetic resonance imaging, nuclear scintigraphy, and contrast echocardiography are the cardiac imaging modalities that have been utilized to validate the imaging of small animals and have adapted to study obesity based cardiac injuries^27^. In vivo magnetic resonance imaging is a tool to provide clear anatomical views of the heart and can be further used to assess accurate heart functions. Cine-MRI is used to obtain the following functional parameters including left ventricular mass, ejection fractions, and cardiac output. The small size and fast heartbeat rate of mice really challenge the accuracy of echographic studies on cardiac functions but still, the availability of high-frequency ultrasound transducers can help to overcome these limitations.

In this study, SiNW-FET device was fabricated using the top-down methodology. The main advantage of this study is that the fabricated device is used as a biosensor for cTnI detection in vivo. The surface of SiNW-FETs was modified to immobilize cTnI antibody for the capture of cTnI antigen. The electrical conductivity, anti-interference ability, and overall biosensor performance were studied. This study aimed to determine cTnI levels in vivo using ECG, MRI, and SVC sampling methods and Syrian hamsters as experimental animal model for the first time.

## Results and discussion

### Design and characteristics of SiNW-FET device

**Figure 1:**
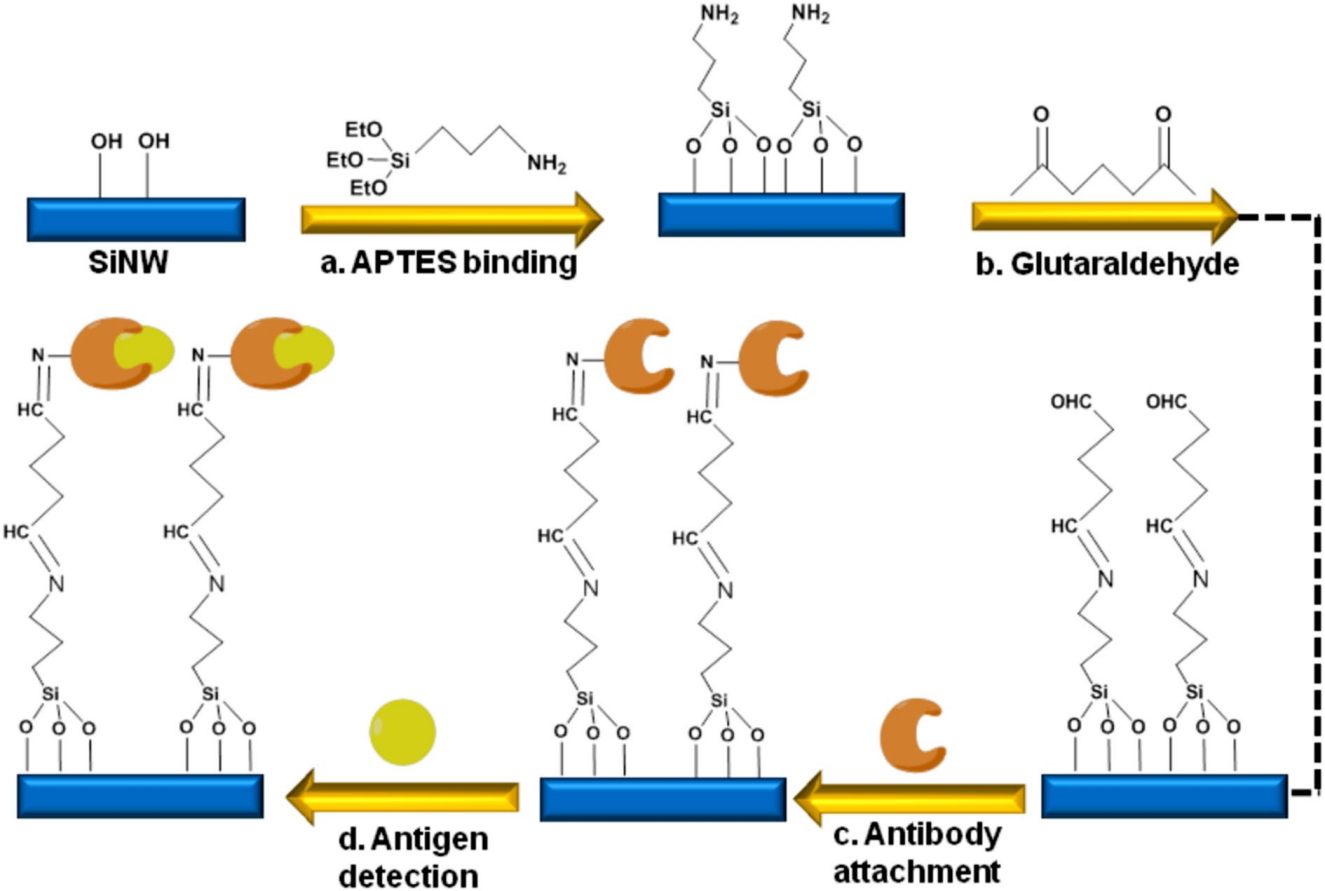
Schematic representation of the chemical process involved during surface modification of SiNW devices: (**a**) APTES addition to SiNW-FET. (**b**) Addition of a bifunctional linker glutaraldehyde. (**c**) Antibody attachment. (**d**) Antigen detection.

SiNW-FETs were designed using the top-down methodology and resulted in invariable dimensions with morphological features in bulk production. These sensors could provide profit-oriented nanodevices in the practical utilities. Figure 1 showed a schematic representation of chemical process involved in the surface modification.

**Figure 2:**
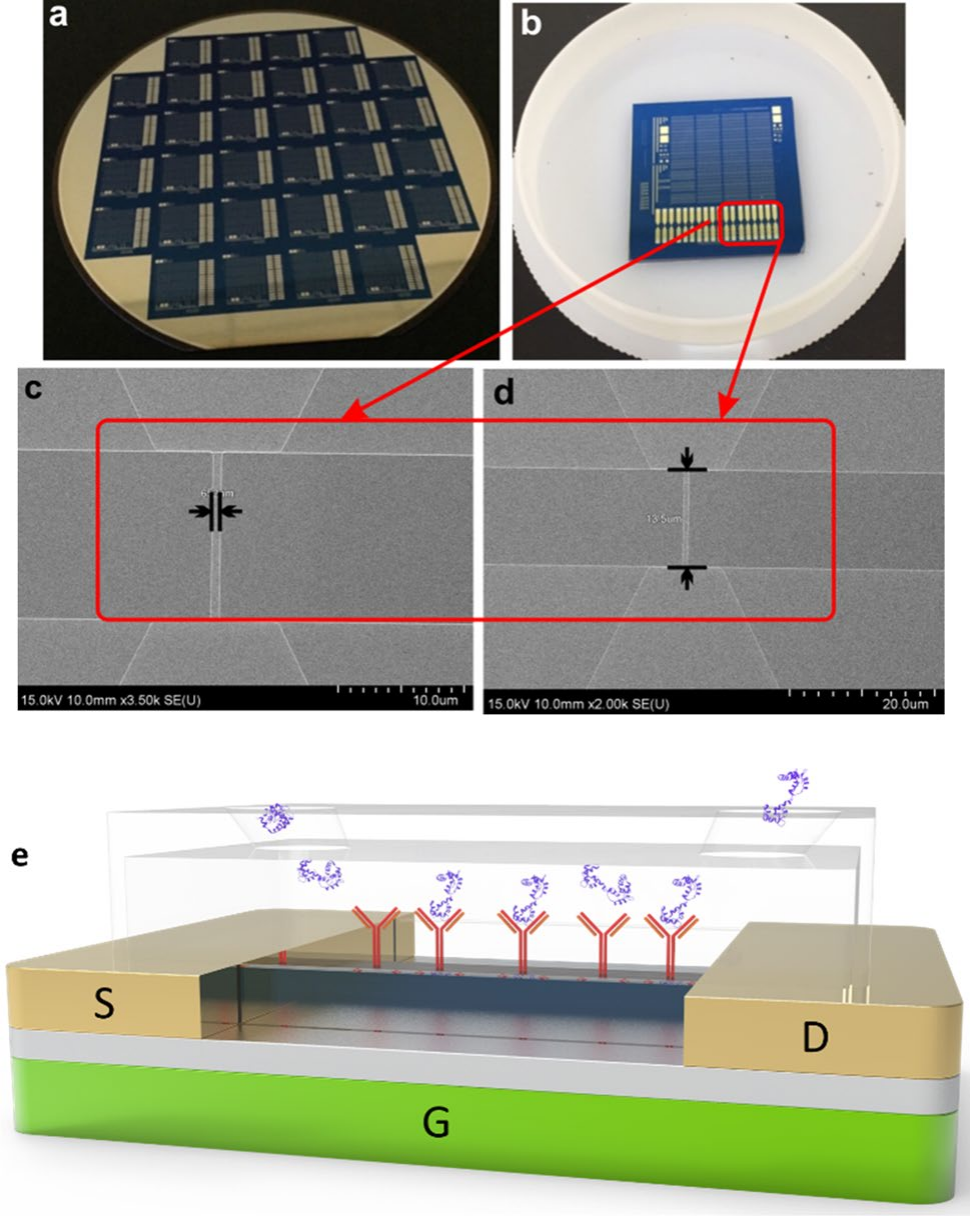
(**a**,**b**) The images of the fabricated SiNW-FET device. (**c**,**d**) showed the SEM images of the representative nanowire region with average length and width. (**e**) Schematic diagram of single SiNW-FET device.

In this study, detection of cardiac cTnI was achieved using MOS-compatible SiNW-FETs by immobilizing cTnI antibodies on the surface of SiNW after fabrication process as shown in Fig. 2. SEM images of SiNW-FET were recorded to account for the morphological features of the device. The images of SiNW-FET device were shown in Fig. 2a,b. Figure 2c,d showed a detailed SEM image and the representative nanowire region. The SiNW-FET showed good alignment and shape and was highly uniformed with an average length of 13.5 µm and width of 652 nm. SEM–EDS method was adopted for the identification of chemical composition on the surface of SiNW-FET (Table S1). Figure 2e represents the schematic diagram of single SiNW-FET device.

### Electrical characteristics of SiNW-FET

The electrical measurements including output behavior of SiNW-FET were presented in Fig. S1 with the gate voltages ranging from 1 to 16 V. In this study, the source meter was used to confirm the current stability of wafer and also to assess material lattice defect caused by instability of signal. The accuracy of flow trend was noted by changing the voltages from 1 to 16 V by observing their current variations. The results showed that high voltage supplies increased the current intensity and indicated that SiNW-FET functioned well as a conducting material. At higher voltage, the curve was disturbed due to the destruction of the SiNW-FET structure which caused defects and unstable outputs. While the defect occurred around 16 V, supply in low voltage still kept the stable circumstances. The observed currents ranged from 4 × 10^–9^ to 7 × 10^–5^ A for different voltages (2, 4, 8, and 16 V). However, the nanowire signals were not generated when the currents were greater than 2.1 × 10^–4^ A. Overall, electrical characteristics have been tested and 0.7 V was applied for all the following biosensing measurements.

### Current response to chemical modification on SiNW-FET

Current changes were recorded after the chemical modification of the SiNW-FET sensor. The completion of the modification process was confirmed by measuring the current change at 0.7 V and the current intensity of the blank chip was found to be 1.14 × 10^–9^ A (Fig. S2). Further, FESEM was used to confirm the surface modification on SiNW-FET. The FESEM is an advanced research grade SEM with excellent performance for surface imaging. Figure S3 showed the FESEM images of APTES- and glutaraldehyde-modified SiNWs and their elemental compositions are given in Tables S2 and S3.

### Debye screening

The Debye length is one of the important parameters capable of tuning the device performance. Debye lengths recorded for PBS buffer at different concentrations were displayed in Fig. S4. It is well known that the ionic strength is inversely proportional to Debye length and current intensity^28^ and a large difference in the current signal can be noticed when using low ionic strength buffer solution. Current measurements of cTnI (0.5 ng/mL) in 0.01 × PBS, 0.1 × PBS, 0.5 × PBS, 0.8 × PBS, and 1 × PBS were carried out to study the effect of different ionic strengths of PBS on cTnI antigen detection. A decrease in Debye length was observed when PBS was increased from 0.01 × to 1 × . This result indicates that upon increasing the buffer solution concentration, the changes in current response becomes small and therefore reduces the sensitivity. In this study, the lowest Debye length accompanied with larger current change and high sensitivity for the sensor device was achieved when using 0.01 × PBS, which was used in the following experiments.

### Detection of human cTnI antigens

**Figure 3:**
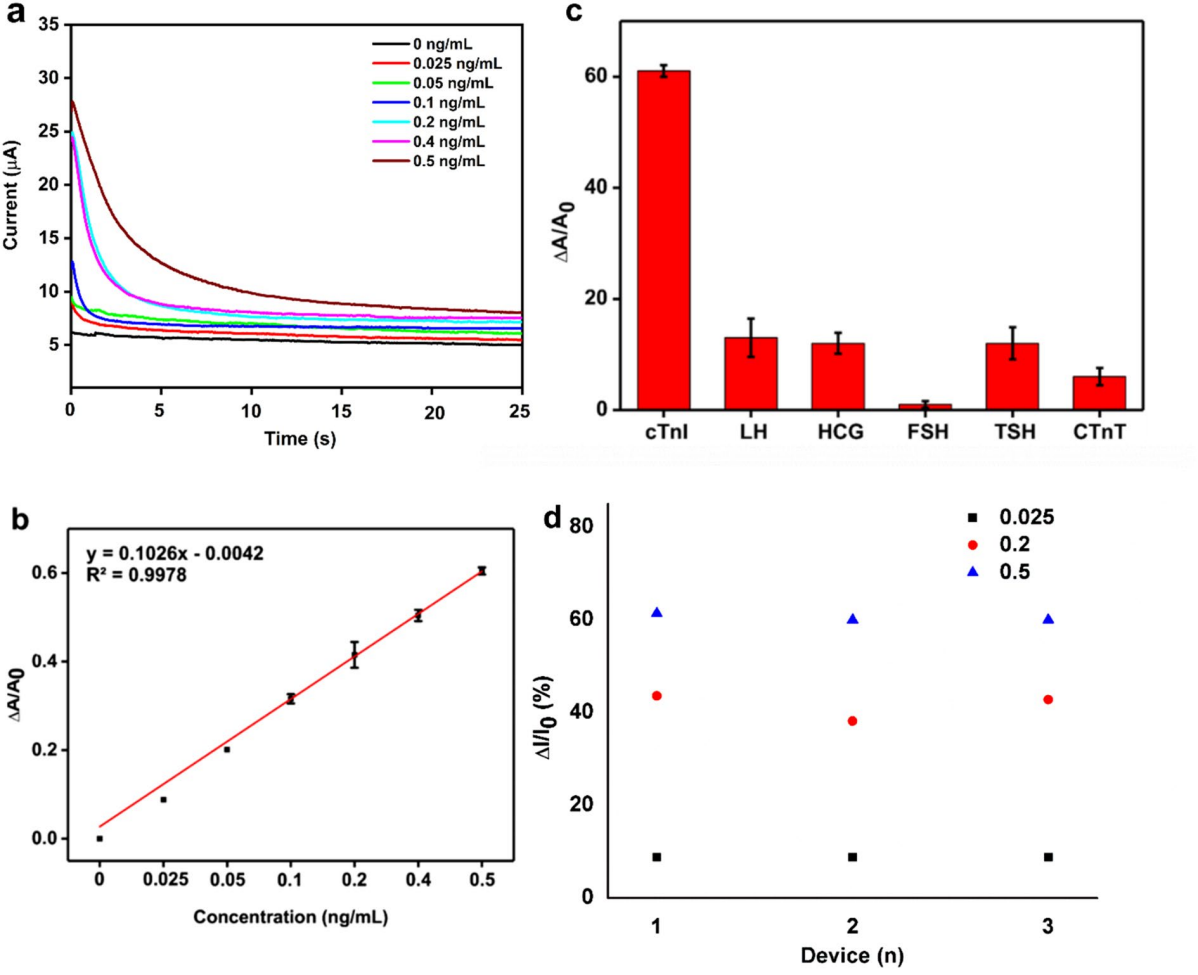
(**a**) Conductance of SiNW-FET immobilized with anti-cTnI for the detection of cTnI at various concentrations. (**b**) Calibration curve of 3a. (**c**) Non-specific binding of LH, HCG, FSH, TSH, and cTnT on the functionalized SiNW-FET surface, determined by the current changes. (**d**) The electrical response of a three sensor chips obtained from three different batches to detect three different cTnI concentratios (0.025 ng/mL, 0.2 ng/mL and 0.5 ng/mL).

Detection and distinction of cTnI antigen at different concentrations in 0.01 × PBS buffer solution were studied in order to generate a new SiNW-FET array chip for detection of cTnI at ultra-low concentrations in biological samples. An appropriate amount of pure cTnI antigen was prepared in 1 × PBS solution as a stock solution and this stock solution was diluted to prepare cTnI antigen at various concentrations by serial dilution. The detection of cTnI antigen at various concentrations in 0.01 × PBS solution was displayed in Fig. 3a. The biosensor displayed a stable and constant response when PBS alone was used as a blank solution and revealed the excellent stability of SiNW-FET device and its whole measurement system. After the stable reading with 0.01 × PBS, cTnI antigen at various concentrations was added and a decrease of conductance was observed with the increased cTnI concentrations. It was very clear that the conductive property of SiNW-FET device is very sensitive in the presence of cTnI. These results clearly indicated that the large difference in current response in the antibody bounded SiNW-FET device was due to the specific binding of cTnI antigens onto cTnI antibodies. Upon each addition of cTnI, a rapid reduction in the signal was observed and the current became stable within 5–10 s. It is hypothesised that the initial drop in signal is related to our chip becoming stable in the depletion regime^29^. The SiNW-FET sensor reached a steady state in 10 s after the binding events have occurred between the cTnI antibodies and antigens, and maintained up to 25 s. The SiNW-FET exhibited high efficacy and fast response time when reacted with cTnI antigens.

The calibration curve was plotted using the different results obtained from the reactions of cTnI antibodies, and the calibration curve with its R^2^ value = 0.9978 was shown in Fig. 3b, suggesting this SiNW-FET device can be used as an effective tool for the accurate and rapid diagnosis of myocardial injury. The current change was noticed when particular concentration was loaded on the chip. There exist only few seconds delay between antigen addition and current change. The biosensor reached a steady state current within 5 s (Fig. S5) once the binding event occurred between cTnI protein and its antibody. Table S4 summarized the comparison of results, materials, and methods of proposed SiNW-FET with other reported cTnI biosensors. From Table S4, the fabricated device in this study showed lower detection limit and broader linear range for cTnI detection compared with the reported biosensors. The MCM-41 mesoporous material for detection of cTnI showed a broad detection range of 0.8–5.0 ng/mL with 0.5 ng/mL detection limit^30^. Using superparamagnetic particles, linear range of 0.03–6.5 ng/mL and 0.03 ng/mL (LOD) was achieved by Dittmer et al.^31^. On the other hand, functionalized gold nanoparticles showed a low detection limit of 0.002 ng/mL with high detection range^32^. Using silicon nanowire, linear range of 0.092–46 ng/mL and 0.092 ng/mL (LOD) was achieved by Kong et al.^23^. The developed SiNW-FET biosensor in this study showed the detection range of 0.025 to 0.5 ng/mL and a detection limit of 0.016 ng/mL, which were better than the reported materials. For the first time, this study aimed to determine the potential thresholds of cardiac magnetic imaging, ECG, and SVC parameters measured early after obesity based cardiac injuries. So far, in the literature, no combination of in vitro and in vivo studies were used to determine the cTnI levels for obesity based cardiac injuries. This is the first report subjected to use Syrian hamsters as experimental mouse models to determine cTnI levels in vivo using ECG, MRI, and SVC sampling methods which are the advantages of this work. Apart from these advantages, care must be taken to improve the device sensitvity upon creating new SiNW-FET device and modifying the fabrication protocols in the future.

### Selectivity and reproducibility

To study the non-specific binding of hormones on the functionalized SiNW-FET surface, control experiments were performed and real-time responses were noted. Five different glycoproteins luteinizing hormone (LH), human chorionic gonadotropin (HCG), follicle stimulating hormone (FSH), thyroid stimulating hormone (TSH), and cTnT were applied to SiNW-FET to investigate the selectivity of the device for cTnI, by injecting 0.5 μg/mL of them into the microfluidic channels. Figure 3c showed the current changes after cTnI, LH, HCG, FSH, TSH, and cTnT flew through the modified SiNW-FET. LH, HCG, FSH, TSH, and cTnT were reduced sequentially by more than 70%, whereas cTnI still retained the highest current signal. These results revealed the specificity of SiNW-FET biosensor towards cTnI, not other tested glycoproteins, and suggested the possibility of capturing cTnI antigens in the presence of other glycoproteins. The reproducibility of the FET sensor is an important parameter for practical applications. Therefore, the fabricated devices from the three different batches (1, 2 and 3) were tested in detecting 0.025 ng/mL, 0.2 ng/mL and 0.5 ng/mL cTnI and the results are shown in Fig. 3d. The results demonstrated that all the tested devices obtained from different batches exhibited closely similar current responses indicating the good reproducibility of the devices for cTnI detection.

### Physiological analysis of the cardiovascular disease induced syrian hamsters

Animal studies were performed using Syrian hamsters. The animals were fed with a controlled diet and body weights were measured every three days for 54 days (Fig. S6). While the weights of normal group Syrian hamsters were increased after taking normal diet for 54 days, their weight gain was 18% less than the fat group.

### Electrocardiogram

**Figure 4:**
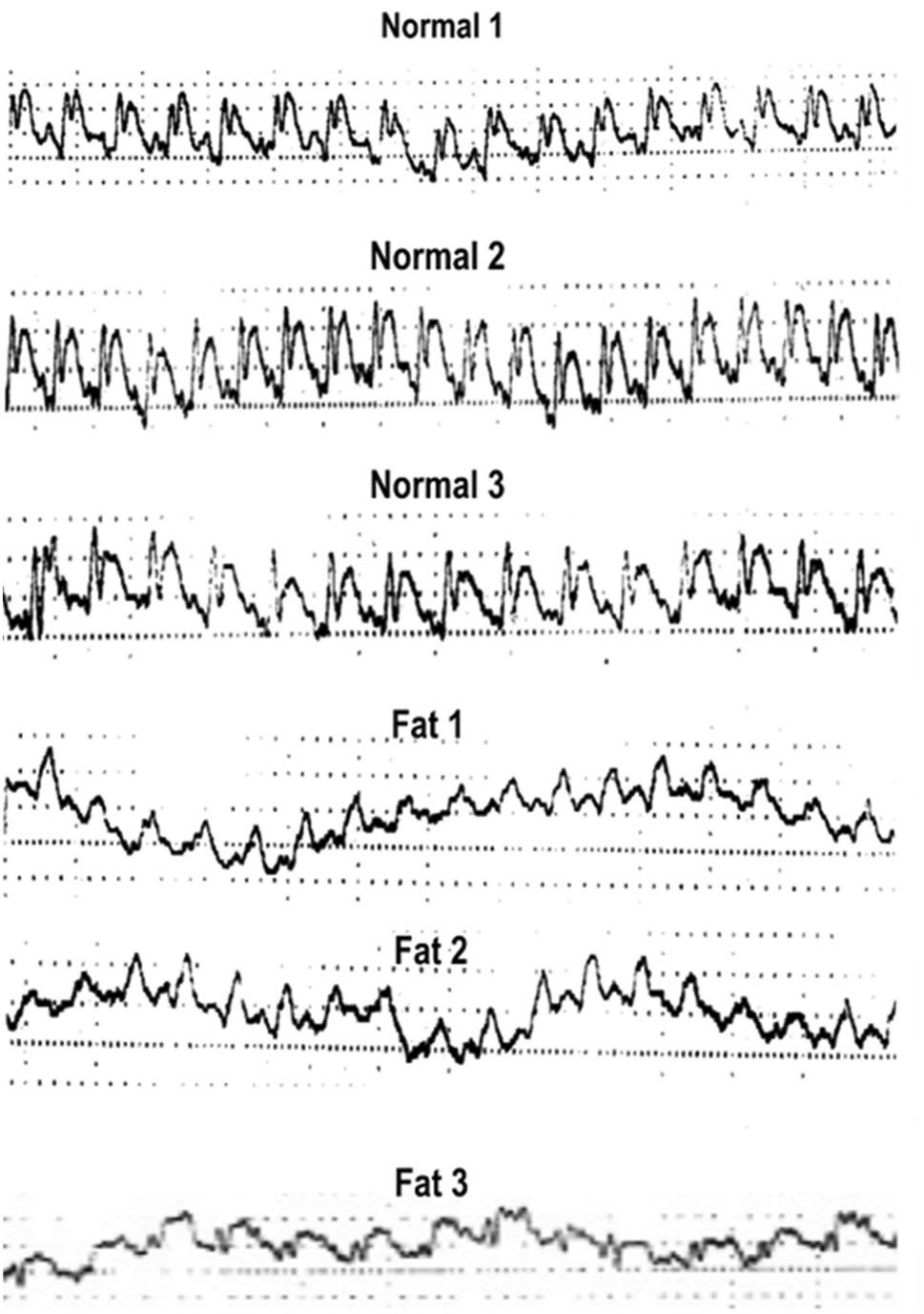
The ECG results of Syrian hamsters.

Major information for the cardio-related diagnosis can be provided by some devices that are able to record the physiological signals produced by the human body. An ECG is a process of measuring the electrical activity of the heart for the diagnosis of heart disease or other malfunctions. The ECG results of Syrian hamsters were shown in Fig. 4.

For ECG measurement, a pair of electrodes are fixed on the body surface with the help of ECG leads to measure the electrical change-depolarization caused by the contraction of muscles. The ECG leads are imaginary lines appeared between the ECG electrodes. Each lead represents the activity related to electrical changes obtained from the heart muscles in a different angle and resulting in electrical images to present different amplitudes and shapes based on electrode position on the body surface. This further allows seeing the heart functions from different angles. In total, 12 ECG leads are available which are classified into 2 major groups as limb leads and chest leads. Limb leads are further classified into 3 (I, II, and III) bipolar limb leads. In this study, the electrodes were fixed on the left hand (Lead III), left leg (Lead II and III), and right hand (Lead II and I) to record ECG. This method generates a sequence of positive and negative waveforms known as PQRST waves. The P and QRS complex waves indicate ventricular and atrial depolarizations, respectively. Differences in these waveforms help to identify the cardiac malfunctions. Animal-induced myocardial infarction model was presented in Fig. 4, which showed different waveforms for normal group and fat group. Typical ECG wave series and their corresponding waveforms generated by the study model was also shown. The ECG results demonstrated a stable PQRST waveform for the normal group. The fat group showed continuous, more rapid and wide QRS waveform which is presumed to be Bundle branch block (BBB), a type of conduction problem caused by this subclinical myocardial injury and may cease to conduct electrical impulses appropriately, and leads to a loss of ventricular synchrony and makes the prolonged ventricular depolarization followed by a corresponding drop in cardiac output. Figure S7 showed the analysis of ECG using the square grid line. Higher squares were observed for the normal group when compared to the fat group. These results showed that normal group has higher fluctuation than that of the fat group.

### Magnetic resonance imaging analysis

In order to assess the cardiac output, heartbeat was recorded for a myocardial injury-induced animal model in both normal and fat groups by continuous motion imaging in the heart using coronal view mobilization and the images were imported to perform Matlab programming according to the selected region of interests. In this study, Matlab has been used as a programming language to analyze 10 dynamic images with the time interval of 0.026 s and labeled time (s) in x-axis and velocity (cm/s) in the y-axis and to obtain the time-velocity curve. The time-velocity curve was used to calculate the forward blood flow and backward blood flow according to the region of interest. Matlab syntax used to calculate the blood flow difference between the normal and fat groups was shown in Fig. S8.

**Figure 5:**
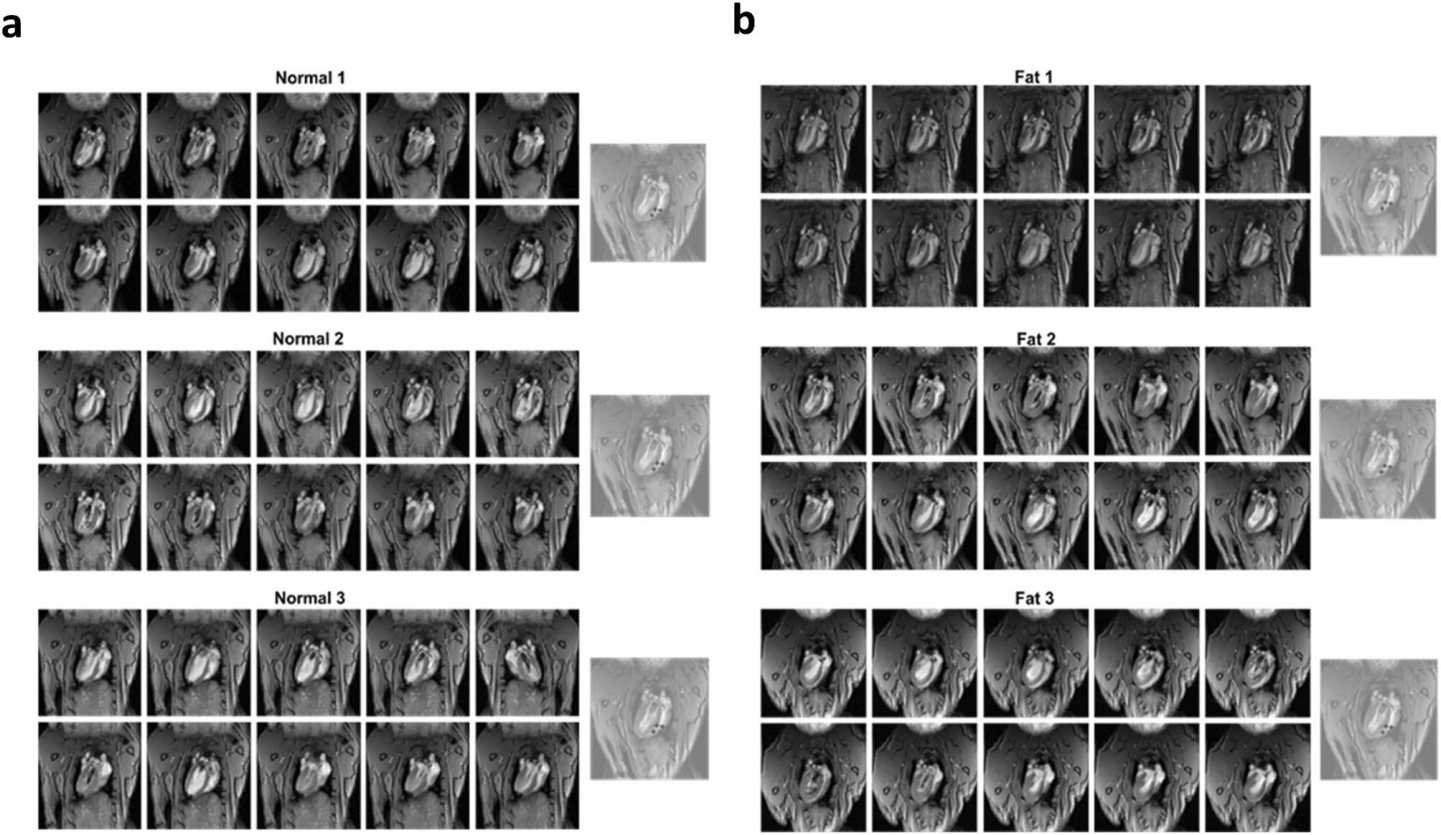
(**a**) Magnetic resonance imaging for normal group Syrian hamsters. (**b**) Magnetic resonance imaging for fat group Syrian hamsters. Column at the right end showed corresponding ROI images.

Magnetic resonance imaging for normal group and fat group Syrian Hamsters was shown in Fig. 5a,b. The animation of the first column from the left-hand side was cut into 10 images and was displayed with a coronal view. The long repetition of time (TR) and the short time of echo (TE) caused the blood to have a bright signal and the images showed different brightness due to the variation in blood flow. ROI was selected at the left ventricle and shown in column 2. The difference between forward flow and backward flow for normal 1, normal 2, and normal 3 hamsters were calculated and the values were 0.0022, 0.0024, and 0.0032, respectively. Similarly, the difference between forward flow and backward flow for fat 1, fat 2, and fat 3 hamsters were also calculated and the values were 0.0011, 0.0015, and 0.0014, respectively. Analysis of the above results showed that the normal group had higher blood flow than the fat group (Table S5), indicating a greater forward blood flow than backward blood flow. This also means that the former blood flow volume is greater than that of inflow blood volume in normal group of hamsters. All these results demonstrated that the cardiac output of the normal group was better than that of the fat group. The blood flow values of normal and fat groups were given in Table S5. According to ECG and MRI imaging analyses respectively, BBB was occurring in the fat group and a worsened cardiac output due to intraventricular contractile dyssynchrony was unveiled in the fat group. In addition, forward blood flow was higher than the backward blood flow in the normal group but was opposite in the fat group. Measurement of cTnI antigen in the blood using SiNW-FET.

### Superior vena cava

After ECG and MRI analyses, Syrian hamsters were subjected to undergo Superior Vena Cava (SVC) blood samplings. The blood from the superior vena cava of the normal and the fat groups was dropped on the SiNW-FET to assess the concentration of cTnI (Fig. S9 and Table S6). The cTnI (%) was calculated using the following equation

$$ {\text{cTnI}}\;\left( \% \right) = \left\{ {\left( {{\text{cTnI}}\;{\text{modified}}\;{\text{chip}} - {\text{blank}}\;{\text{chip}}} \right)/{\text{cTnI}}\,{\text{modified}}\;{\text{chip}}} \right\} \times {1}00 $$

The observed results indicated that the normal group showed a lower percentage of cTnI concentration when compared with the fat group.

### Comparison of proposed SiNW-FET with electrochemiluminescence immuno assay (ECLIA) in cTnI analysis

In addition, the commercially available ECLIA was performed to determine the cTnI value and compared with the values obtained from the proposed SiNW-FET (Table S6). Both the proposed SiNW-FET and ECLIA exhibited similar results for cTnI determination.

In this work, a major effort was to fabricate SiNW-FET biosensor for the rapid detection of cTnI, a cardiac biomarker for myocardial injury. This device achieved the electrical detection of cTnI with fast reaction time, high sensitivity and specificity with a detection limit of 0.016 ng/mL. This is the first report to use Syrian hamsters as the animal model to compare normal diet (normal group) and high-fat diet (fat group) on body weights, cardiac function by ECG and MRI, and cTnI, a myocardial injury biomarker using a newly designed SiNW-FET sensor. The proposed SiNW-FET device was compared with the standard clinical laboratory method ECLIA in the determination of blood cTnI levels in hamsters, and compatible values for cTnI were observed with both methods. For its stability and lowest detection limit, this newly developed SiNW-FET device may provide a potential sensing platform for rapid screening of cTnI in early diagnosis of acute myocardial infarction.

## Experimental section

### Materials

Human cardiac troponin I (cTnI, 98%), human cardiac troponin T (cTnT, 98%), sodium periodate (NaIO_4_, 98%), sodium hydroxide (NaOH, 99%), hydrogen chloride (HCl, 12 N), (3-aminopropyl)triethoxysilane (APTES), glutaraldehyde, luteinizing hormone (LH, 98%), human chorionic gonadotropin (HCG, 98%), follicle stimulating hormone (FSH, 95%), thyroid stimulating hormone (TSH, 98%), and polydimethylsiloxane (PDMS, 99%) were purchased from Sigma Aldrich (MO, USA). All the other reagents and chemicals used in this study were of analytical grade and were used as received without any purification unless specified.

### Methods

The 6-inch P-type prime wafer with 150 ± 0.5 mm diameter, 675 ± 25 µm thickness, 15 ~ 25 Ω cm resistance with 57.5 ± 2.5 mm primary flat was obtained from Wafer Works Corporation (Hsinchu, Taiwan). The microfluidic syringe pump was ordered from Chemyx- fusion100 (Texas, USA). PDMS elastomer kit was purchased from Sil-More Industrial Ltd (New Taipei City, Taiwan). X-ray photoelectron spectra (XPS) data were obtained by using ESCALab 220i-XL electron spectrometer from National Nano Device Laboratories (Hsinchu, Taiwan). The base pressure used was 3 × 10^–9^ mbar. Adventitious carbon contamination was used as a charge reference for the interpretation of XPS spectra. Scanning Electron Microscope (SEM) images with Energy Dispersive Spectroscopy (EDS) were taken using Hitachi SU-8010 + EDS Oxford MAX150 instrument to observe the morphology of samples. Multi-functional Field-Emission Scanning Electron Microscopy images (FESEM) with Electron Back-Scattered Diffraction (EBDS) observation were recorded using JSM-7800F PRIME + EBSD NordlysMax3 (JEOL, USA). Transmission Electron Microscopy (TEM) images were taken using TECNAI 20 (Philips, Netherlands) instrument. Current measurements on the reaction of SiNW-FET with cTnI were measured by source measurement (Keithley 2614B). Electrocardiogram (ECG) was recorded using CX 50, Compact Xtreme Ultrasound System (Philips, Netherlands). Magnetic Resonance Imaging (MRI) analyses were performed in the Department of Electrical Engineering, Magnetic Resonance Imaging Laboratory (National Taiwan University, Taiwan). Continuous cardiac imaging of Syrian hamsters was performed using 7 T Bruker S-300 Biospec/ Medspec MRI (Bruker, USA) instrument. Java-TSI Fast IV recorder (Oracle, USA), Microsoft Export Document Series (Microsoft, USA), Horos Imaging Analysis Application (Annapolis, USA), and Matrix Laboratory (MATLAB) (The MathWorks, USA) were the software used in magnetic resonance imaging and the image analyses.

### Preparation of cTnI antibody and cTnI antigen

The cTnI antibody (1 ng/mL) was prepared by dissolving cTnI antibody (1 ng) in 1 mL of 0.01 × PBS. Sodium azide (0.02%) was added to the cTnI antibody solution and stored at − 20 °C. Similarly, cTnI antigen solution (1 ng/mL) was prepared by dissolving cTnI antigen (1 ng) in 1 mL of 0.01 × PBS. Sodium azide (0.02%) was added to the cTnI antigen solution and stored at − 20 °C. Both cTnI antibody and antigen stock solutions were further diluted to prepare working solutions with 0.025, 0.05, 0.1, 0.2, 0.4, and 0.5 ng/mL concentration.

### Fabrication of SiNW-FET device

In this study, metal–oxide–semiconductor (MOS) technology developed at National Device Laboratory (NDL) was used for the fabrication of SiNW-FET device. The biosensor was fabricated on a silicon-on-insulator (SOI) wafer with a diameter of 150 ± 0.5 mm, a thickness of 675 ± 25 µm, and buried oxide layer. The H-wet-oxide and H-nitride oxidative layers with a 300 Å thickness were coated on top of SOI wafer to form an oxidation layer. Anisotropic wet etching with buffered oxide etches were used to etch SiNWs and the surface was washed with sulfuric acid and hydrofluoric acid to get the patterns. Ion implantation of boron was performed to obtain an effective contact region. For diffusion, diffusion furnace was used to create the patterns using gold by metallization process in a low-pressure chemical vapor deposition chamber. Finally, a stack of nitride-oxide-nitride passivation layer was formed to hold the metal wire against contamination. High-resolution FE-SEM was used to identify the structure of the SiNW-FET and further measured length/width of the fabricated SiNW-FET.

### Surface modification on SiNW-FET

In this study, the surface of SiNW-FET was chemically modified in order to allow the cTnI antibody-antigen binding event to take place after device fabrication. To start with, SiNW arrays were submerged in a solution of 2% APTES in a mixture of ethanol/water in the ratio of 95:5 (v/v %) for about 2 h, as reported in the previous literature^33^. The attachment of APTES molecules on the hydroxyl terminated silicon oxide surface of the nanowire resulting in an amine-terminated surface. The array chips were then immersed in a 2.5% glutaraldehyde in water solution for 1 h. This is followed by the attachment of cTnI antibody by applying the cTnI antibody solution in phosphate buffered solution (PBS) for 2 h at room temperature and further washed with the same buffer solution. Addition of glutaraldehyde, a cross-linker, forms a covalent bridge between the amine functionalized nanowire surface and an amine group of a biomolecule. To prevent non-specific protein binding during detection, passivation was applied to remove the unreacted aldehyde moieties by applying 100 mM ethanolamine solution in PBS for about 1 h and washed again using the same buffer solution. Figure 1. represents the schematic representation of the chemical process involved during surface modification of SiNW devices.

### Signal measurement of cTnI antigens

Direct assays were performed in all the binding studies. Pure human cTnI antigen was diluted to the desired concentrations including 0.025, 0.05, 0.1, 0.2, 0.4, and 0.5 ng/mL in PBS solution. Real time electrical sensing of cTnI was measured by monitoring the changes in conductance of the functionalized device in response to different concentrations of cTnI.

To perform conductance measurements, the voltage from drain to source (V_ds_) was biased at fixed voltage 0.7 V. The experimental protocol includes (1) curve of current across drain (I_d_) and back gate (substrate) voltage (V_bg_) was firstly recorded using 0.01 × PBS solution (pH 7.4) as the background curve; (2) various concentrations of cTnI antigen (0.025, 0.05, 0.1, 0.2, 0.4, and 0.5 ng/mL) in PBS buffer were injected at a flow rate of 0.2 mL/s and incubated for 10 min to link the antibody and antigen; (3) unbound cTnI sample was removed by 0.01 × PBS washing; (4) I_d_ − V_bg_ curve was measured with 0.01 × PBS as environmental solution; (5) the same experimental protocol was repeated for cTnI at the mentioned concentrations to obtain corresponding I_d_ − V_bg_ curves.

### The reaction of SiNW-FET with cTnI and current measurements

PDMS kit was used to construct the microfluidic system. Materials A (antibody) and B (antigen) were taken in the ratio of 10:1 for 1 mL. After mixing, 0.9 mL was added into the microfluidic system. The vacuum in the system was sucked out to release the air bubbles for 30 min, heated for 3 h at 110 °C, and finally cooled down. All the electrical measurements were performed in PBS buffer solution at pH 7.4 and room temperature. Different concentrations of cTnI antigens prepared by diluting with PBS buffer was added on nanowires, kept it for 10 min, then washed using distilled water and dried under a nitrogen atmosphere. The electrical signals were monitored after the addition of cTnI antigens with antibody functionalized SiNW-FET.

In this study, the surface of fabricated SiNW-FET was scanned to measure the current by applying voltages in the range of 0.0 V to 1.0 V using source meter. The antibody-immobilized SiNW-FET was placed in the microfluidic system and an air stream pump was used to fix the chip on measuring platform of source meter. Probes were attached to source and drain electrode of the chip and step size voltages from 0.00 to 1.00 V were supplied. The sample was injected at the rate of 0.2 mL/s by syringe pump into the microfluidic system with gate voltage 0.7 V. Real time detection of cTnI in serum condition was studied by monitoring the current changes with respect to various cTnI concentrations in different time intervals. For the non-specific binding test, 0.5 ng/mL concentrations of LH, HCG, FSH, and TSH were prepared and the same incubation time was used for bio-sensing experiments. Current changes were measured with antibody-immobilized SiNW chip to study the effects of repeated use. In this experiment, wafer modification procedure is repeated every time before current measurement.

### Debye length measurement

Charge screening has great influence on FET biosensors due to the formation of strong ionic bonds between the electrons of materials and atoms of the proteins, thus altering electrical conductance. The Debye screening length has a direct relationship with the screening effect. Screening length is defined as the distance between immobilized target moiety and outer sensing area separated by a screen of counter electrons that are further attracted towards the nanowire known as the "electrical double layer". The Debye length in a buffer solution can be calculated using the formula λ_D_ = √(εK_b_T/q^2^N_d_), where ‘ε’ is the dielectric constant, ‘K_b_’ is the Boltzmann’s constant, ‘T’ is the absolute temperature in kelvins, ‘q’ is the elementary charge, and ‘N_d_’ is the density of dopants. Charge screening is directly related to Debye length and longer Debye length indicates less charge screening from the SiNW. Increase in ionic strength reduces the Debye charge screening, which further reduces the device sensitivity. Also, the Debye length will be increased by using diluted buffer solutions. However, desirable dilution leads to very low ionic strengths and may affect the biological activity of the antigens or proteins. Since the ionic concentration is dependent on charge screening effect, selecting an accurate buffer solution with suitable Debye screening length is necessary to avoid any jeopardies in the data collection^23,24^. To study the Debye screening effect, PBS buffer solutions with different ionic strengths of 0.01 × PBS, 0.1 × PBS, 0.5 × PBS, 0.8 × PBS, and 1 × PBS were prepared using deionized water.

### Animal studies

Syrian hamsters (female) were purchased from BioLASCO Taiwan Co., Ltd. Animal studies were carried out in accordance with all provisions associated with care and handling of animals used in experiments, as specified by ethical law in Taiwan. Control group hamsters were fed with the normal diet, while the fat group hamsters were fed with high fat diet, obtained from BioLASCO Taiwan Co., Ltd. All the experiments were performed in accordance with institutional guidelines. The procedures of animal handling in this study were reviewed and approved by the Ethical Committee of National Chiao Tung University.

### Acute myocardial infarction model

Syrian hamsters were used as the animal model to conduct all the following studies including ECG, MRI, and SVC. The hamsters were divided into two groups as normal and fat groups. Hamsters in normal group were fed with general (mfg) diet, while hamsters in fat group were fed with high-fat diet. Both groups were maintained by a controlled feed of about 6–7 g/day. Body weights were measured every three days until 54 days. After body weight measurement, ECG was recorded to check the heartbeat. Changes in PQRST waveforms were noted and the differences between normal group and the fat group were compared. After ECG, all the Syrian hamsters were subjected for MRI analysis to calculate the cardiac output. The continuous cardiac imaging was achieved by using the following conditions: Multi-slice intra-gate flash sequence, pilot scan view, TR = 72 ms, TE = 29 ms, FOV = 35 mm, matrix size = 256 × 256, slice thickness = 0.7 mm, 10 movie frames (move phase). In addition, Matlab programming was used to analyze the Syrian Hamster's left ventricular image and the region of interest (ROI) was also selected for all the tested mice. The data were entered into Matlab programming to evaluate the cardiac outputs by comparing the forward and backward blood flows of all the hamsters.

### Superior vena cava (SVC) blood sampling

Each hamster was anesthetized and blood was collected from superior vena cava (SUV), between the sternum and the first rib, following a previous report^34^. The collected blood was transferred into the heparinized tube to avoid coagulation. Blood was then dropped on SiNW-FET and the cTnI concentrations in both groups were determined.

## Supplementary Information

Supplementary Information
