# Supplementary material for: Utilization of silicon nanowire field-effect transistors for the detection of a cardiac biomarker, cardiac troponin I and their applications involving animal models
Source: Sci Rep. 2020 Dec 16;10:22027. doi: 10.1038/s41598-020-78829-7 (PMC7745037; doi:10.1038/s41598-020-78829-7)
Supplement: Supplementary file 1 — Supplementary Information [file 41598_2020_78829_MOESM1_ESM.docx]

**Utilization of silicon nanowire field-effect transistors for the detection of a cardiac biomarker, cardiac troponin I and their applications involving animal models**

**Shih-Mein Chang,^1,†^ Sathyadevi Palanisamy,^1,†^ Tung-Ho Wu,^2,†^ Chiao-Yun Chen,^3,4^ Kai-Hung Cheng,^5^  Chen-Yi Lee,^6^ Shyng-Shiou F. Yuan^7,8,9,^* and Yun-Ming Wang****^1,10,^***

^1^Department of Biological Science and Technology, Institute of Molecular Medicine and Bioengineering, Center for Intelligent Drug Systems and Smart Bio-devices (IDS2B), National Chiao Tung University, 75 Bo-Ai Street, Hsinchu 300, Taiwan

^2^Division of Cardiovascular Surgery, Department of Surgery and Division of Surgical Critical Care, Department of Critical Care Medicine, Veterans General Hospital, Kaohsiung 813, Taiwan

^3^Department of Radiology, Faculty of Medicine, College of Medicine, Kaohsiung Medical University, Kaohsiung, Taiwan

^4^Department of Medical Imaging, Kaohsiung Medical University Hospital, Kaohsiung, Taiwan

^5^Division of Cardiology, Department of Internal Medicine, Kaohsiung Medical University Hospital, Kaohsiung, Taiwan

^6^Department of Electronics Engineering, National Chiao Tung University, Hsinchu, Taiwan

^7^Translational Research Center, Kaohsiung Medical University Hospital, Kaohsiung Medical University, Kaohsiung, Taiwan

^8^Department of Obstetrics and Gynecology, Kaohsiung Medical University Hospital, Kaohsiung Medical University, Kaohsiung, Taiwan

^9^Faculty and College of Medicine, Kaohsiung Medical University, Kaohsiung, Taiwan

^10^Department of Biomedical Science and Environmental Biology, Center for Cancer Research, Kaohsiung Medical University, Kaohsiung 807, Taiwan

**^†^**These three authors contributed equally

*Corresponding authors:

Yun-Ming Wang

Tel: +886-3-5712121 ext. 56972

Fax: +886-3-5729288

E-mail: [ymwang@mail.nctu.edu.tw](mailto:ymwang@mail.nctu.edu.tw)

Shyng-Shiou F. Yuan

Tel: +886-7-3121101 Ext. 2557

E-mail: yuanssf@ms33.hinet.net

**Fig. S1.** The electrical measurements including output behavior of SiNW-FET were presented with the gate voltages varied from 8V to 16 V.


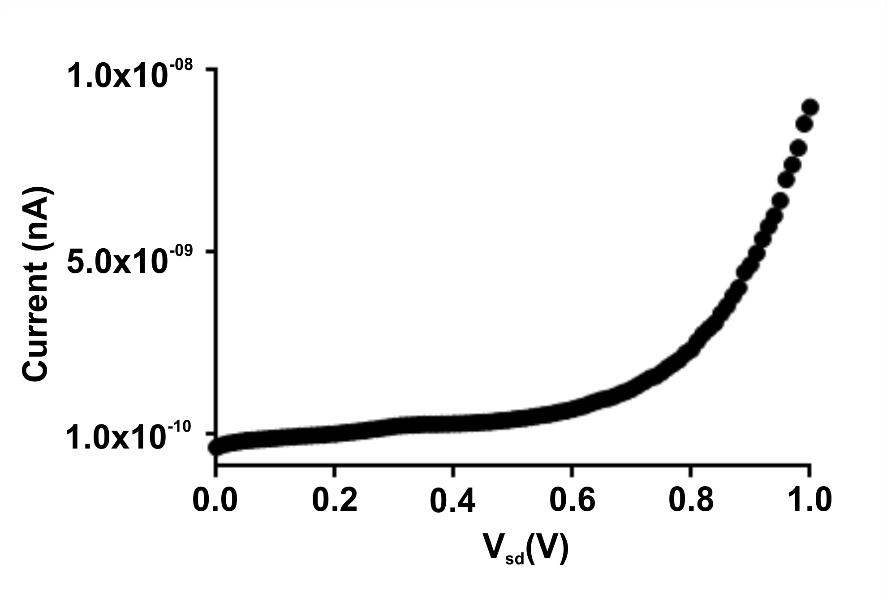


**Fig. S2.** The basic current intensity of SiNW-FET. Increasing the source to drain voltage caused a simultaneous current rise.


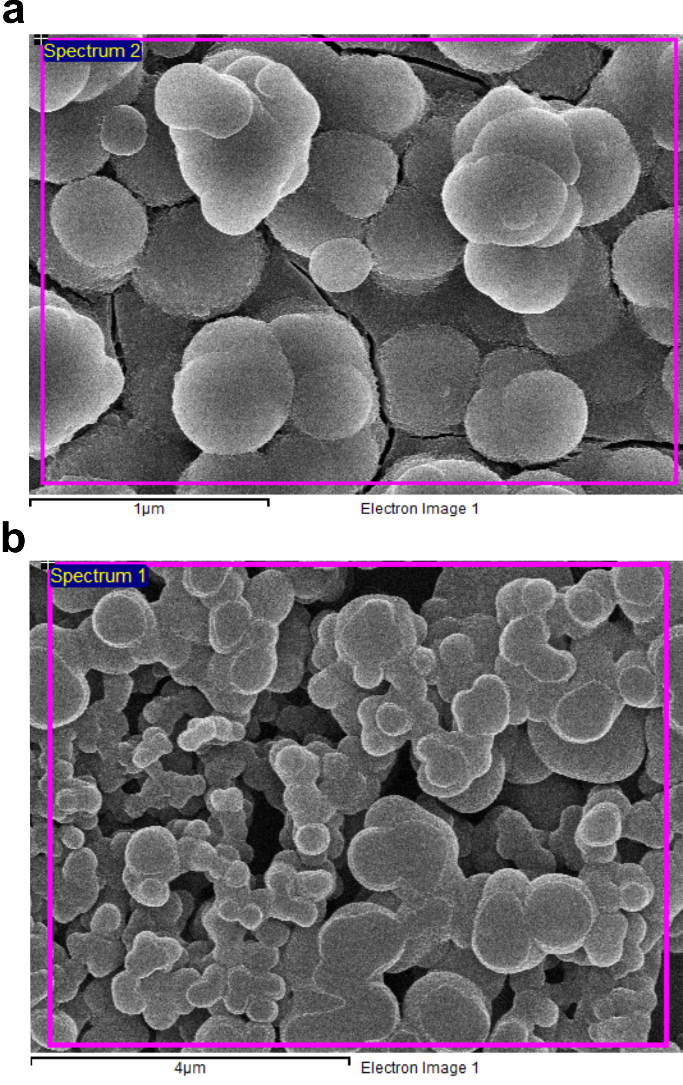


**Fig. S3.** (a) FESEM image of APTES-modified SiNW; (b) FESEM image of glutaraldehyde-modified SiNW.


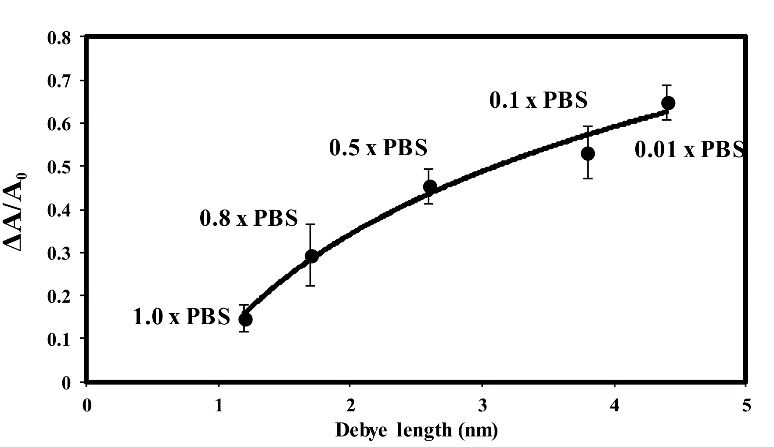


**Fig. S4.** The influence of different ionic strengths of PBS in SiNW-FET on Debye length measurement.


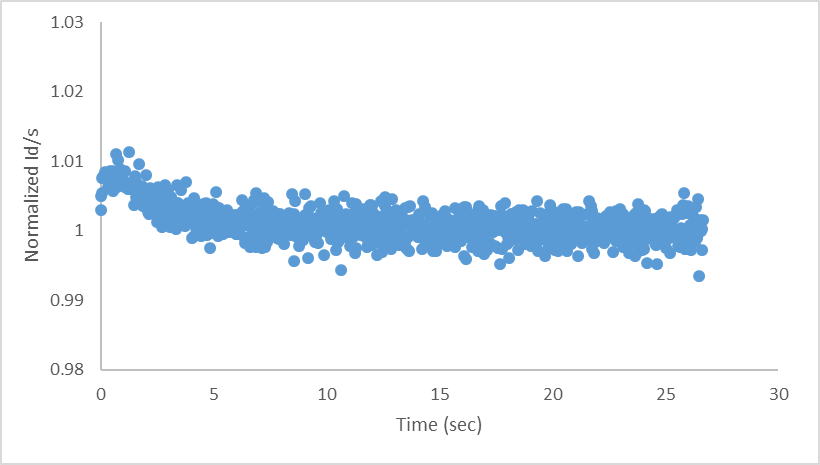


**Fig. S5.** Normalized I_ds_ upon addition of 0.016 ng/mL cTnI


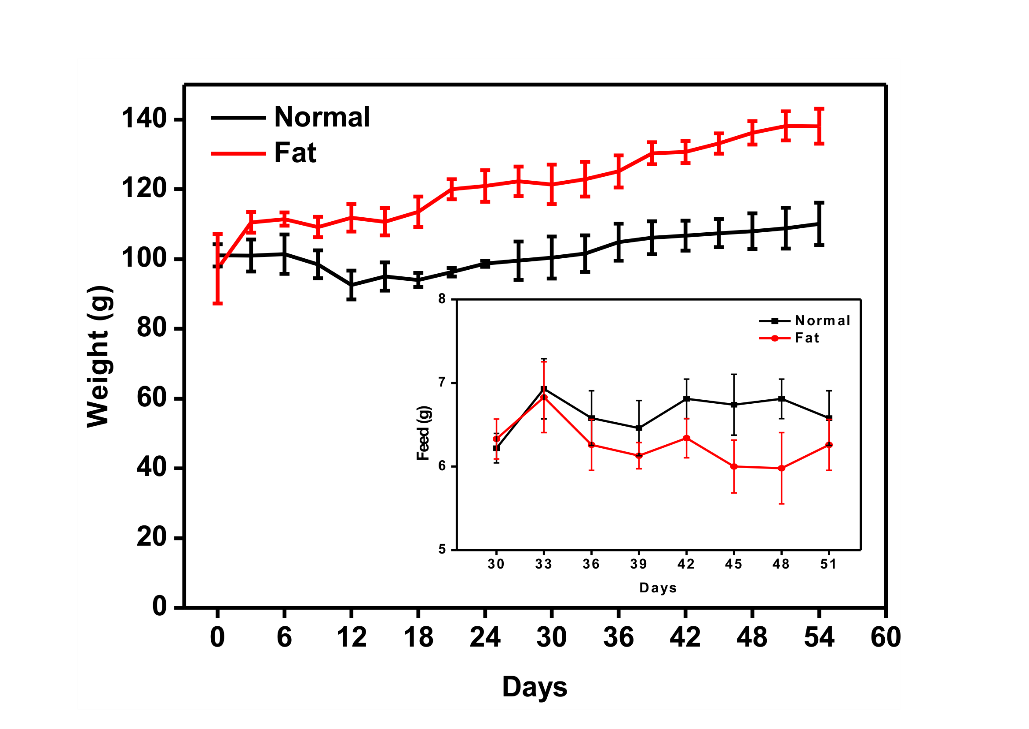


**Fig. S6.** Body weight measurement in normal group and fat group Syrian hamsters.

**
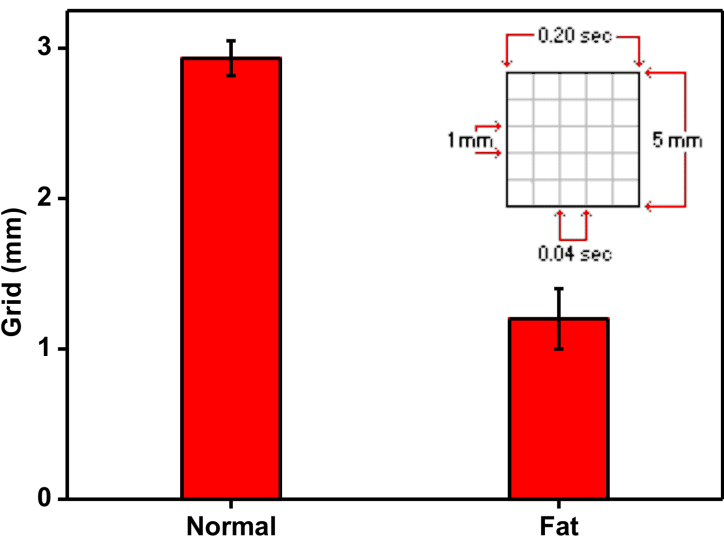
**

**Fig. S7.** Analysis of ECG square grid.

**
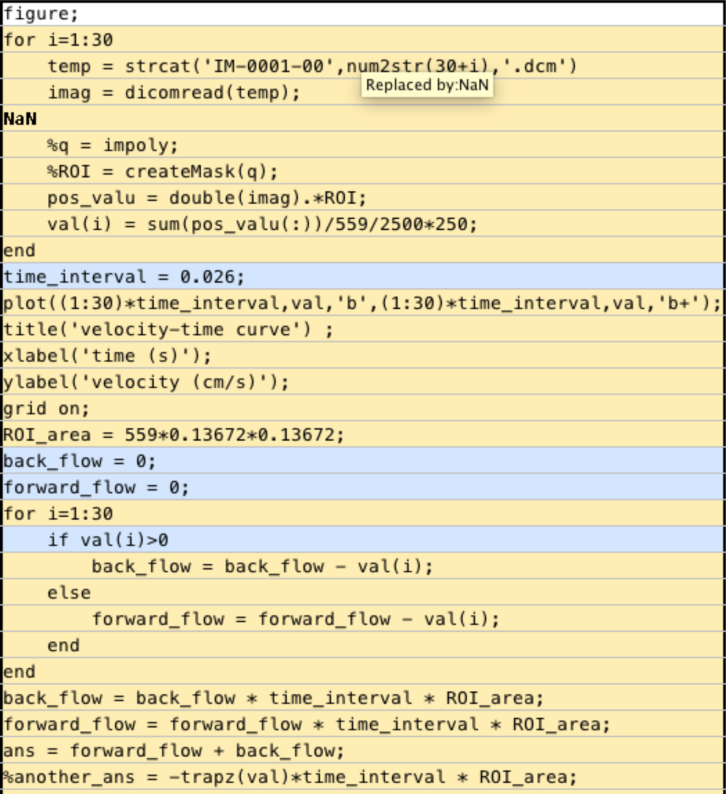
**

**Fig. S8.** Matlab study for the cardiac output analysis.


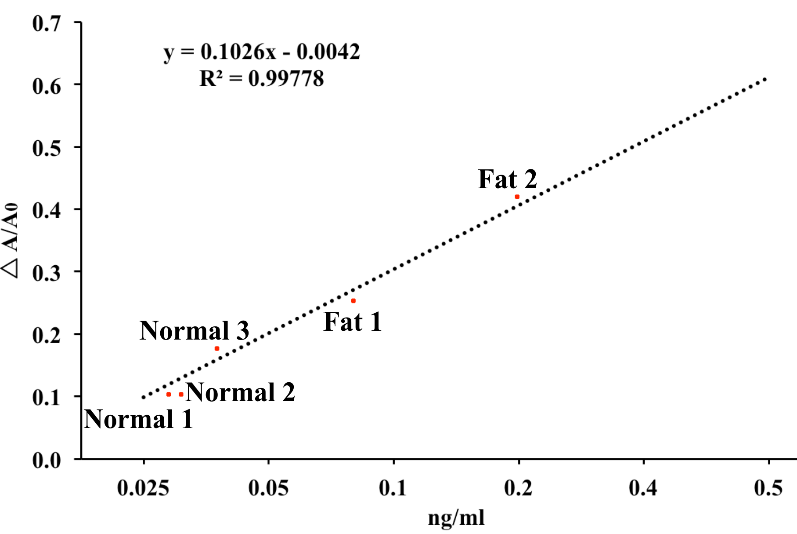


**Fig. S9.** Measurement of cTnI concentration in normal and fat groups Syrian hamsters.

**Table S1.** The chemical composition of SiNW surface

| Element | Weight % | Atomic % |
| --- | --- | --- |
| C | 6.95 | 14.08 |
| O | 12.25 | 18.64 |
| Si | 76.31 | 66.13 |
| Zr | 3.32 | 0.89 |
| Pd | 1.16 | 0.27 |

**Table S2.** The chemical composition of APTES-modified SiNW

| Element | Weight % | Atomic % |
| --- | --- | --- |
| C | 32.82 | 33.28 |
| N | 11.67 | 23.19 |
| O | 32.05 | 31.73 |
| Si | 19.8 | 11.17 |
| Zr | 3.66 | 0.64 |
|  |  |  |

**Table S3.** The chemical composition of glutaraldehyde-modified SiNW

| Element | Weight % | Atomic % |
| --- | --- | --- |
| C | 49.77 | 65.75 |
| N | 15.54 | 15.41 |
| O | 31.75 | 17.94 |
| Si | 1.19 | 0.59 |
| Zr | 1.76 | 0.31 |

**Table S4.** A comparison of the proposed SiNW-FET sensor with other reported materials for the cTnI determination.

| **Methods** | **Materials** | **Linear range (ng/mL)** | **LOD (ng/mL)** | **Reference** |
| --- | --- | --- | --- | --- |
| Electrochemical immunoassay | MCM-41 mesoporous material | 0.8~5.0 | 0.5 | ([Guo et al. 2005a](#_ENREF_11)) |
| Optomagnetic | Superparamagnetic materials | 0.03~6.5 | 0.03 | ([Dittmer et al. 2010](#_ENREF_7)) |
| Electrochemiluminescence immunosensor | Functionalized gold nanoparticles | 0.025~10 | 0.002 | ([Shen et al. 2011](#_ENREF_32)) |
| FET nanodevice | Silicon nanowire | 0.092~46 | 0.092 | ([Kong et al. 2012](#_ENREF_20)) |
| FET nanodevice | Silicon nanowire | 0.025~0.5 | 0.016 | **This study** |

**Table S5.** MRI analysis in Syrian hamsters

| Categories | Normal | Fat |
| --- | --- | --- |
| Blood flow | 2.6×10^-3^ ±0.4% | 1.3×10^-3^ ±0.2% |

**Table S6.** cTnI values read by SiNW-FET and ECLIA assay in clinical laboratory

| Hamster | Normal 1 | Normal 2 | Fat 1 | Fat 2 |
| --- | --- | --- | --- | --- |
| SiNW-FET value (ng/mL) | 0.027 | 0.033 | 0.08 | 0.257 |
| ECLIA assay | 0.031 | 0.042 | 0.12 | 0.256 |
